# Erector Spinae Plane Block and its Impact on Postoperative Diaphragmatic Dysfunction in Morbidly Obese Patients Undergoing Laparoscopic Sleeve Gastrectomy: A Double-Blind Randomized Control Trial

**DOI:** 10.1007/s11695-025-08337-y

**Published:** 2025-11-04

**Authors:** Mina Adolf Helmy, Mohamed Saber Mostafa, Arsany Talaat Saber, Mai Ahmed Ali, Lydia Magdy Milad

**Affiliations:** Cairo University hospitals, Cairo, Egypt

**Keywords:** Erector spinae block, Class III obesity, Diaphragmatic excursion, Diaphragmatic dysfunction

## Abstract

**Background:**

Postoperative diaphragmatic dysfunction (PODD) is a serious sequela following laparoscopic surgery, particularly in individuals with class III obesity. We aimed to evaluate the efficacy of erector spinae plane block (ESPB) in reducing the incidence of PODD and postoperative pulmonary outcomes in individuals with class III obesity undergoing laparoscopic sleeve gastrectomy.

**Methods:**

This randomized controlled trial included 81 participants living with obesity with a body mass index (BMI) greater than 40 who underwent sleeve gastrectomy. Participants were randomly allocated to the control group or the ESPB group. The primary outcome was the incidence of postoperative diaphragmatic dysfunction, defined as mean diaphragmatic excursion (MDE) < 10 mm at 2 h postoperatively. Secondary outcomes included pulmonary function tests, specifically forced expiratory volume at one second, forced vital capacity, and peak expiratory flow rate, as well as pain scores, nalbuphine consumption, and the ROX index. An experienced operator evaluated diaphragmatic excursion, and the mean diaphragmatic excursion was calculated as the average of the right and left hemidiaphragms.

**Results:**

The incidence of PODD was significantly lower in the ESPB group compared to controls (10% vs. 73%). ESPB recipients demonstrated higher diaphragmatic excursion, pulmonary function parameters, and reduced pain scores and opioid consumption. A strong positive correlation (r = 0.786) was found between diaphragmatic excursion and ROX index at 2 h postoperatively.

**Conclusion:**

Bilateral ESP block in individuals with class III obesity undergoing sleeve gastrectomy significantly reduces the incidence of postoperative diaphragmatic dysfunction, preserves pulmonary function, and reduces postoperative nalbuphine consumption. These findings support the integration of erector spinae plane blocks into multimodal perioperative strategies to enhance respiratory outcomes and patient comfort in patients living with obesity undergoing metabolic bariatric surgery.

## Introduction

Class III obesity, defined as a body mass index (BMI) greater than 40 kg/m^2^, is an escalating global health concern with numerous pathological consequences. Metabolic bariatric surgery, particularly gastric volume reduction procedures, has emerged as an effective therapeutic intervention for achieving significant and sustained weight loss. However, these procedures are not without complications, notably challenges in postoperative pain management, respiratory compromise, and diaphragmatic dysfunction [1]. Postoperative diaphragmatic dysfunction, defined as reduced diaphragmatic excursion of less than 10 mm[2], is a clinically significant issue, particularly in individuals with class III obesity. Pain is a primary contributor to postoperative respiratory impairment [2]. Moreover, laparoscopic surgery is known to exacerbate pulmonary dysfunction, with prior studies demonstrating postoperative declines in forced expiratory volume at one second (FEV₁), forced vital capacity (FVC), and peak expiratory flow rate (PEFR) [3]. Diaphragmatic dysfunction in this context is multifactorial, influenced by variables such as capnoperitoneum, patient positioning, impaired cough reflex, and most prominently, pain [2].

Postoperative pain following laparoscopic sleeve gastrectomy arises from both somatic due to port-site incisions and visceral manipulation during gastric resection [4]. Substantial evidence supports the reliability and accuracy of diaphragmatic ultrasound as a tool for assessing diaphragmatic function in intensive care and perioperative settings [5–7].

While regional anesthesia techniques have shown promise in improving postoperative pulmonary outcomes, no prior studies have specifically examined the impact of the erector spinae plane block (ESPB) on diaphragmatic function. We hypothesize that the use of ESPB would reduce the incidence of postoperative diaphragmatic dysfunction in individuals living with class III obesity undergoing laparoscopic sleeve gastrectomy. To our knowledge, this is the first randomized controlled trial to explore this potential association.

## Methods

This randomized controlled trial was conducted at a university hospital, following approval from the institutional research ethics committee (N-152-2025) and registration of the clinical trial (NCT07022600). Eligible participants were adult individuals aged 18–65 years living with class III obesity (BMI > 40) undergoing laparoscopic sleeve gastrectomy. Participants were randomly assigned to one of two groups: the control group (n = 40) or the ESPB group (n = 41). Randomization was performed using an online tool (randomizer.org), and allocation was concealed using sequentially numbered, sealed, opaque envelopes that were opened by an independent research assistant one hour before surgery. Exclusion criteria included: inadequate diaphragmatic ultrasound views, preexisting pulmonary conditions (e.g., chronic obstructive pulmonary disease, bronchial asthma, interstitial fibrosis), contraindications to nerve block, or refusal to participate.

All patients received intravenous ketorolac (30 mg) and paracetamol (1 g) approximately 30 min prior to the induction of anesthesia. Following confirmation of adherence to preoperative fasting guidelines, general anesthesia was induced using intravenous propofol (2.5 mg/kg) and fentanyl (1.5 µg/kg), both dosed according to lean body weight, along with atracurium (0.5 mg/kg) based on total body weight to facilitate endotracheal intubation. Anesthesia was maintained with 1.2% isoflurane in oxygen, supplemented by intermittent boluses of atracurium (0.1 mg/kg) every 20 min to ensure adequate neuromuscular blockade. Prophylactic antiemetic therapy included intravenous ondansetron (8 mg), administered near the end of the procedure. Upon completion of surgery, and while patients remained under general anesthesia, those assigned to the ESPB group received bilateral erector spinae plane blocks under ultrasound guidance. An experienced anesthesiologist (MH), who was not involved in data collection, performed nerve blocks using a Versana Essential ultrasound system (GE Medical Systems Co., Ltd., China). A linear transducer (L6-12-RS, 4–16 MHz) was positioned vertically 2–3 cm lateral to the T8 spinous process. Under aseptic conditions, an 80 mm in-plane needle (Stimuplex; B. Braun, Germany) was introduced in a cranial-to-caudal trajectory. Hydrodissection with 2 mL of normal saline was employed to confirm the correct fascial plane. Thereafter, 20 mL of local anesthetic mixture (10 mL 0.5% bupivacaine, 5 mL 2% lidocaine, and 5 mL saline) was administered on each side.

Following surgery, all participants were monitored for one hour in the post-anesthesia care unit (PACU). A standardized multimodal analgesia regimen was implemented, consisting of intravenous (IV) paracetamol 1 g every 8 h, with the first dose administered 6 h postoperatively, and a single IV dose of ketorolac 60 mg given at the same time point. Pain at rest was evaluated using the Numeric Rating Scale (NRS; 0 = no pain, 10 = worst pain imaginable) at PACU, 1, 2, 6, 12, and 24 h postoperatively. Rescue analgesia with IV nalbuphine (5 mg) was provided for NRS scores ≥ 4. Pulmonary function tests were conducted using the SP80B Spirometer (CONTEC Medical Systems Co., Ltd., China) at baseline (preoperative), and at 2- and 24-h post-block administration. After application of a nasal clip, all participants were instructed to take maximal inspiration followed by a forceful and rapid exhalation. The test was repeated three times, and mean values were recorded for forced expiratory volume in one second (FEV1), forced vital capacity (FVC), and peak expiratory flow rate (PEFR).

Diaphragmatic excursion was assessed using the same ultrasound machine by an experienced operator (LM), with > 60 prior similar assessments, who was blinded to the group allocation. Measurements were taken preoperatively and at 2 and 24 h postoperatively. A curved probe (4 C-RS, 2–5 MHz) was placed horizontally at one of the lower intercostal spaces at the right anterior axillary and left mid-axillary lines to visualize the corresponding hemidiaphragms. M-mode imaging was switched on while the participant was asked to take deep breaths with sweep speed adjusted to zero to allow capturing of multiple breaths in one image, and DE for each side was measured across at least three respiratory cycles as the distance from the highest to the lowest point. The mean diaphragmatic excursion (MDE) was calculated as the average of the right and left hemidiaphragms.

**Fig. 1:**
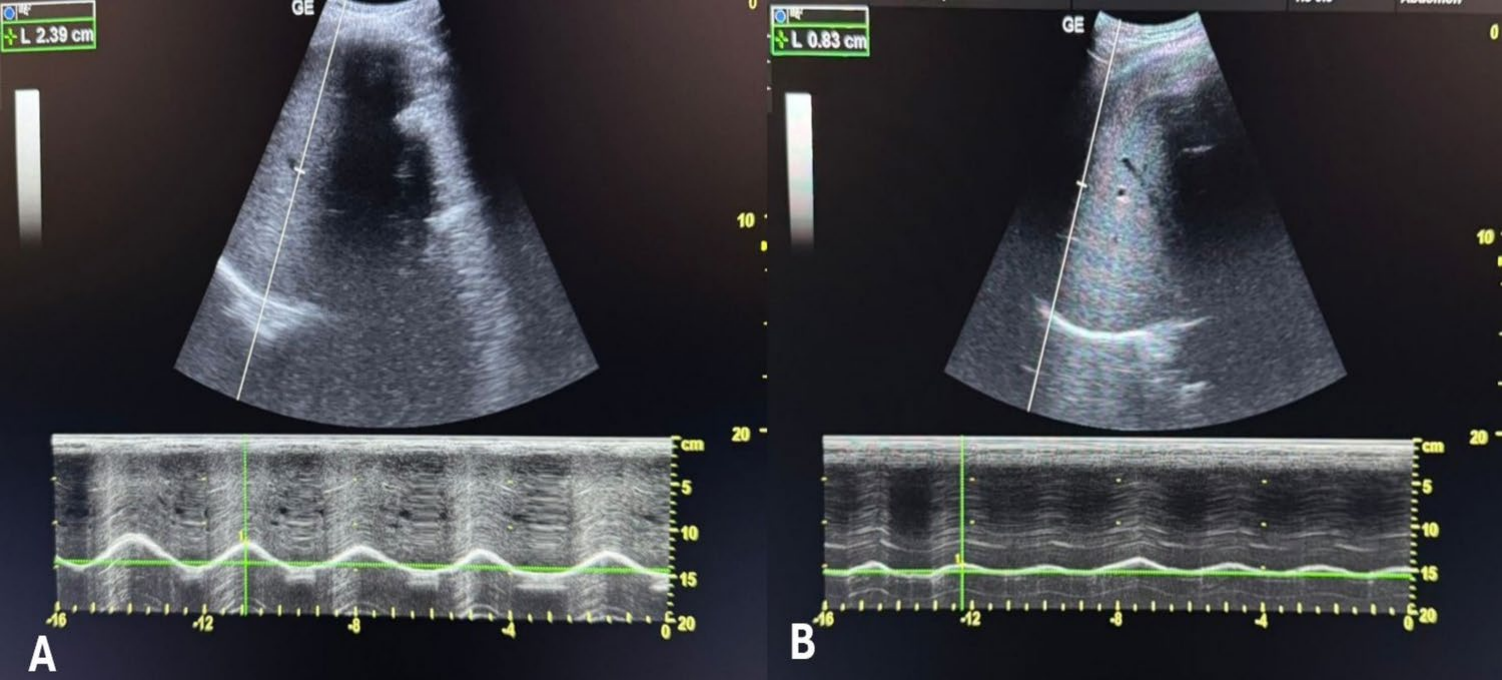
Decreased right diaphragmatic excursion two hours postoperatively in a patient without an erector spinae plane block

The primary outcome was the incidence of diaphragmatic dysfunction (DD), defined as MDE < 10 mm at 2 h postoperatively (Fig. 1). Secondary outcomes included pulmonary function parameters (FEV1, FVC, PEFR), diaphragmatic excursion at all points, total 24-h Nalbuphine consumption, and NRS scores at PACU, 1, 2, 6, and 24 h.

Peripheral oxygen saturation (SpO₂) and respiratory rate (RR) were recorded 2 h postoperatively, corresponding with the timing of diaphragmatic excursion assessment. These values were used to calculate the ROX index using the formula: SpO₂/FiO₂ divided by RR.

## Sample Size

Sample size calculated based on the primary outcome: incidence of PODD. A pilot study involving 10 participants who underwent general anesthesia without ESPB revealed PODD in 80% at 2 h postoperatively. Assuming that a 50% relative reduction in incidence is clinically significant, a sample size of 72 was calculated (α = 0.05, power = 0.95). To account for potential dropouts, the final sample size was adjusted to 40 individuals per group.

## Statistical Analysis

Data normality was assessed using the Kolmogorov–Smirnov test. Non-normally distributed data are presented as medians and quartiles (Q1-Q3) and were analyzed using the Mann–Whitney U test. Normally distributed variables are expressed as mean ± standard deviation and compared using an unpaired t-test. Categorical data are reported as frequencies and percentages and analyzed using Chi-squared or Fisher’s exact tests, as appropriate. Correlation between diaphragmatic excursion and ROX index was evaluated using Spearman’s rank correlation. A p-value < 0.05 was considered statistically significant. To identify independent risk factors for postoperative diaphragmatic dysfunction (PODD), a multivariate logistic regression analysis was performed. Covariates included ESPB group assignment, diabetes mellitus status, age, insufflation pressure, and duration of capnoperitoneum. Variables were selected based on clinical relevance and baseline group differences. Analyses were performed using MedCalc version 19 (Mariakerke, Belgium) and SPSS (version 26) for Microsoft Windows (Armonk, NY: IBM Corp.).

## Results

**Fig. 2:**
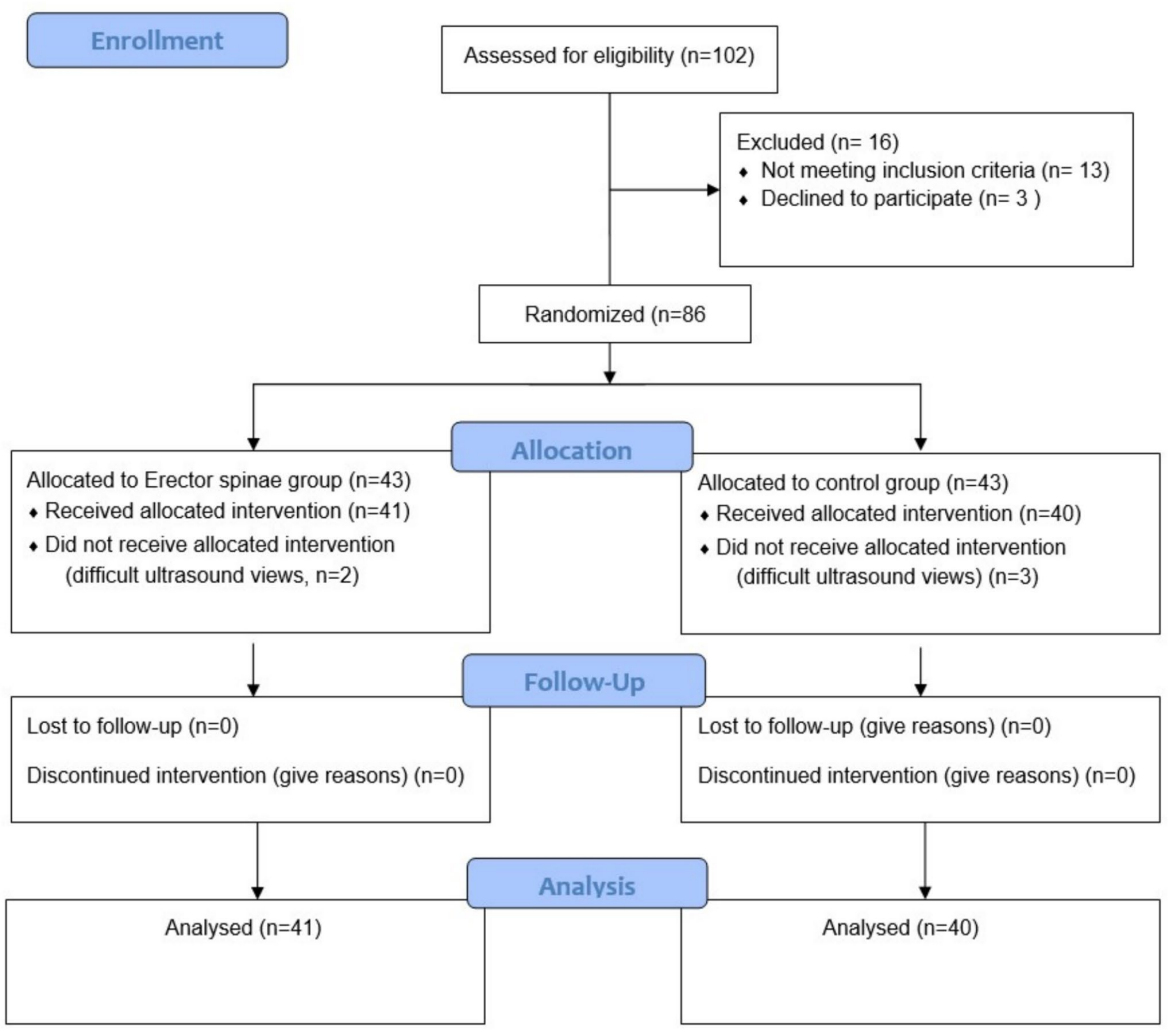
Patient enrollment

A total of 102 individuals were screened for eligibility. Of these, 16 were excluded based on predefined criteria, and 86 were randomized. Five participants were subsequently withdrawn from the study due to suboptimal ultrasound views, resulting in 81 individuals included in the final analysis (Fig. 2). Apart from the difference in diabetes mellitus prevalence, baseline demographic and clinical characteristics, including age, sex, BMI, duration of surgery, duration of pneumoperitoneum, insufflation pressure, and other comorbidities, were comparable between the ESPB and control groups (Table 1). Although the prevalence of diabetes mellitus differed significantly between groups, this variable was included as a covariate in a sensitivity analysis, which confirmed the robustness of the observed associations.

**Table 1:** Baseline and demographic data, data presented as mean $\pm$ SD, median (quartiles), and Count (percentage)

	Control group (n = 40)	ESPB group (n = 41)	P value
Age (Years)	36.5(29–45.8)	39(32–48.5)	0.243
Male gender	18(45)	22(53.7)	0.508
BMI (Kg/m2)	45.8(43.6–48.3)	45.7(43.8–47.9)	0.880
Duration of surgery (minutes)	83 $\pm$ 9	87 $\pm$ 9	0.096
Duration of Capno-peritoneum (minutes)	63 $\pm$ 10	65 $\pm$ 9	0.430
Insufflation pressure (cmH2O)	13(11–13)	12(11–13)	0.464
Comorbidity, n			
HTN	24/40 (60%)	25/41 (61%)	0.928
DM	15/40 (38%)	5/41 (12%)	0.017*
IHD	6/40 (15%)	4/41 (10%)	0.704
Hypothyroidism	6/40 (15%)	4/41 (10%)	0.704
AF	1/40 (3%)	0	0.990

^*^Denotes statistical significance

AF: atrial fibrillation, BMI: body mass index, DM: diabetes mellitus, HTN: hypertension, IHD: ischemic heart disease

**Fig. 3:**
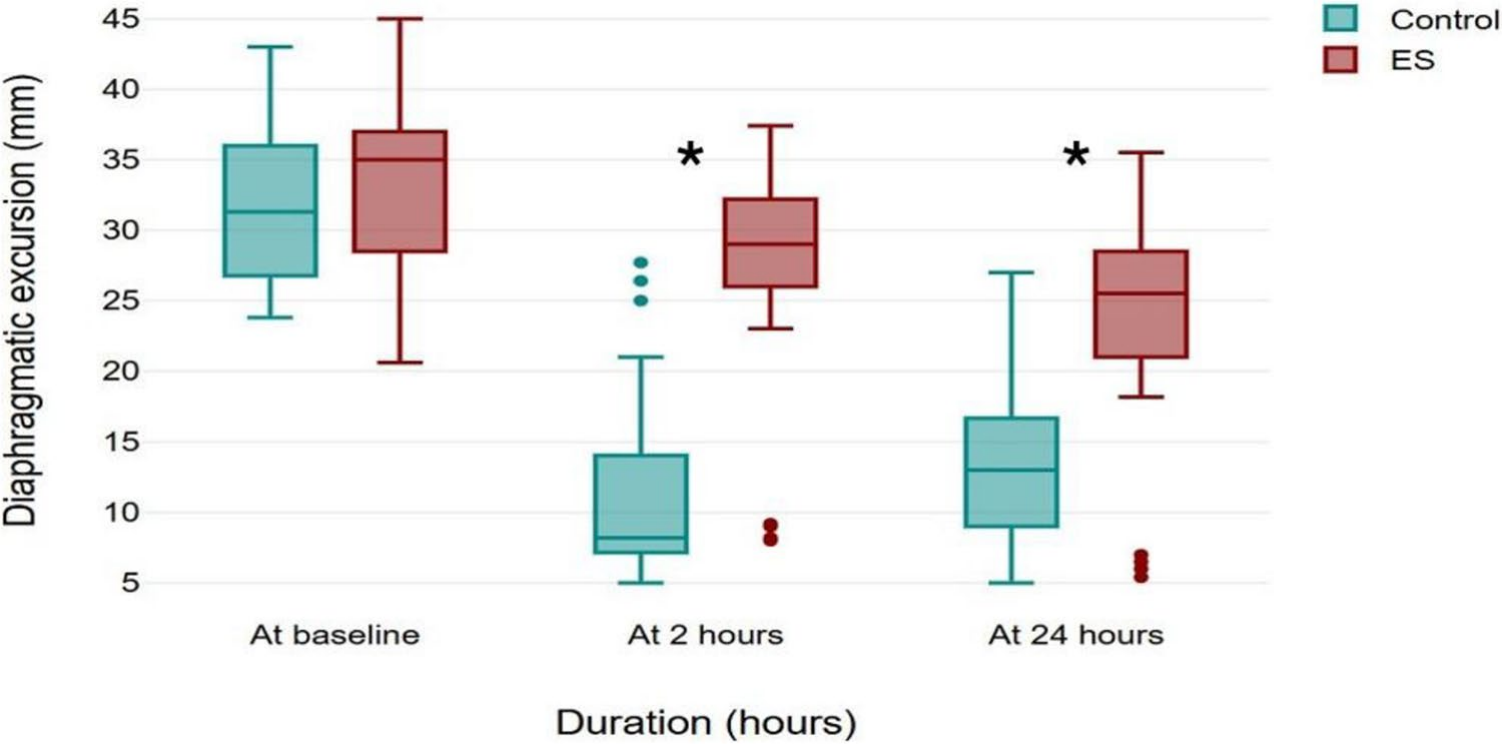
Diaphragmatic excursion over time, data presented as median (quartiles). DE: diaphragmatic excursion, ES: erector spinae. *: Denotes statistical significance

**Fig. 4:**
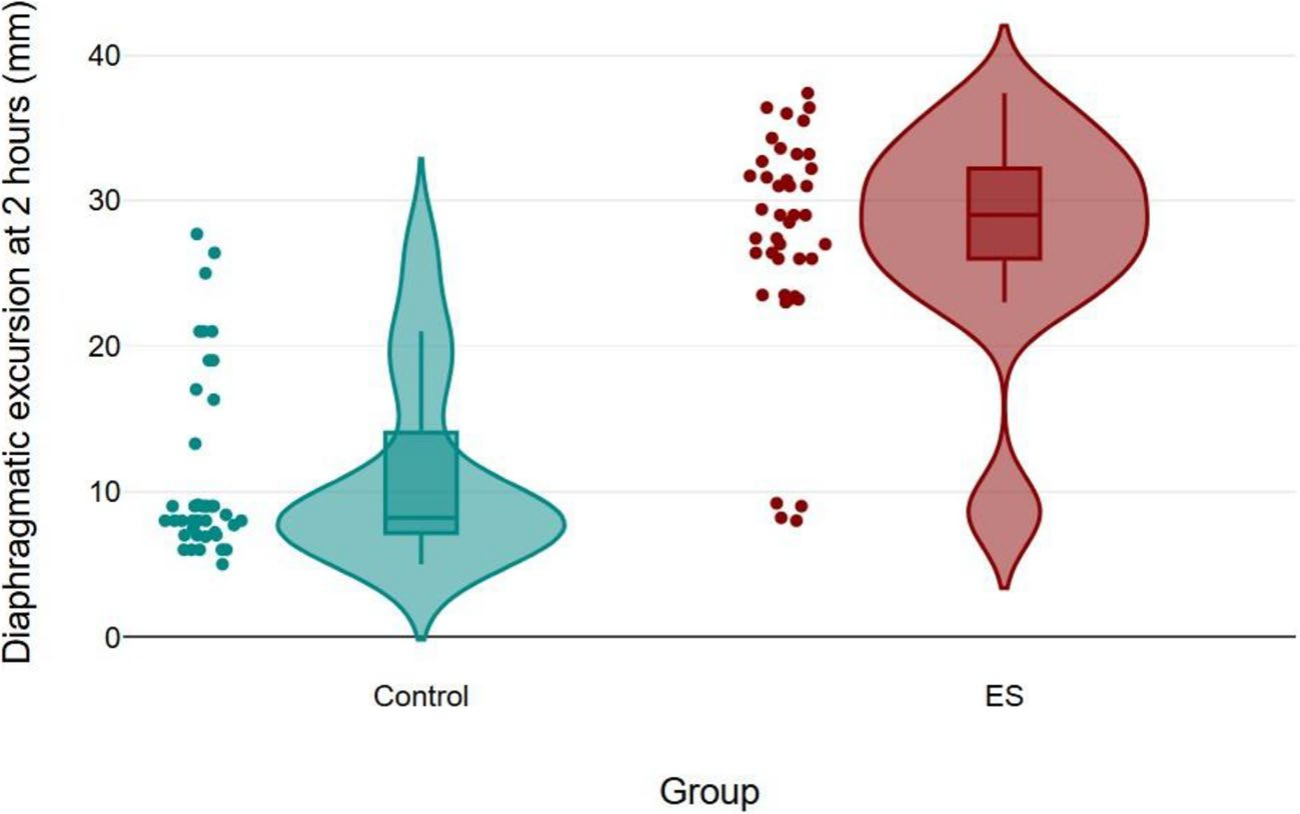
Box-violin plot with individual data points for both groups. ES: erector spinae

The incidence of diaphragmatic dysfunction at 2 h postoperatively was significantly lower in the erector spinae plane block group compared to the control group (10% vs. 73%, respectively). Diaphragmatic excursion decreased postoperatively in both groups; however, the magnitude of reduction was notably greater in the control group (Table 2; Figs. 3, 4).

**Fig. 5:**
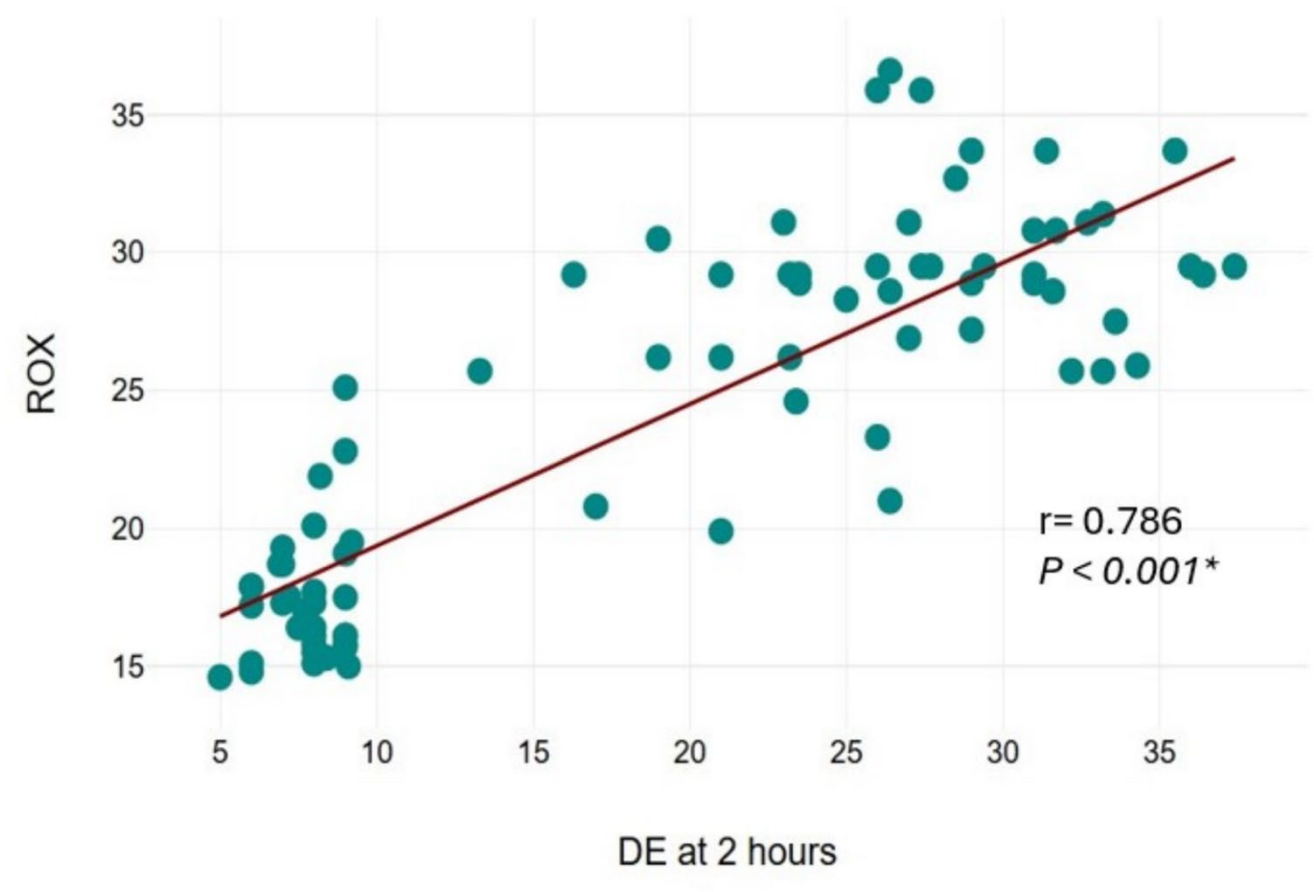
Correlation between diaphragmatic excursion at 2 h after surgery and ROX index. DE: diaphragmatic excursion, R: correlation coefficient

A strong positive correlation was observed between diaphragmatic excursion at 2 h and the ROX index, as determined by Spearman’s rank correlation analysis (correlation coefficient: + 0.786, 95% CI: 0.686–0.857; p < 0.001) (Fig. 5).

Pulmonary function test parameters, including FEV1, FVC, and PEFR, were significantly higher in the ESPB group compared to the control group at postoperative assessments (Table 2).

**Table 2:** Ultrasound and pulmonary functions

	Control group (n = 40)	ESPB group (n = 41)	P value
MDE (mm)			
Baseline	31.3(26.3–36)	35(28.3–37.2)	0.275
2 h Postoperative	8.2(7.1–15.6)	29(24.8–32.5)	< 0.001*
24 h postoperative	13(9–16.9)	25.5(20.5–28.9)	< 0.001*
FEV1 (L)			
Baseline	2.9(2.7–3.3)	3.1(2.8–3.3)	0.342
At 2 h	2.3(1.9–2.6)	2.7(2.4–3.1)	< 0.001*
At 24 h	2.3(1.9–2.6)	2.6(2.3–2.9)	0.010*
FVC (L)			
Baseline	3.5 $\pm$ 0.52	3.7 $\pm$ 0.53	0.127
At 2 h	2.9 $\pm$ 0.54	3.4 $\pm$ 0.5	< 0.001*
At 24 h	3 $\pm$ 0.52	3.2 $\pm$ 0.53	0.032*
PEFR (L/S)			
Baseline	6.2 $\pm$ 0.95	6.1 $\pm$ 1.25	0.500
At 2 h	5 $\pm$ 0.92	5.7 $\pm$ 1.19	0.006*
At 24 h	5.1 $\pm$ 0.91	5.5 $\pm$ 1.22	0.099

^*^Denotes statistical significance

FEV1: forced expiratory volume at one second, FVC: forced vital capacity, MDE: mean diaphragmatic excursion, PEFR: peak expiratory flow rate

Additionally, individuals who received ESPB blocks demonstrated superior respiratory profiles, including greater diaphragmatic excursion and ROX index values, along with lower respiratory rates, 24-h nalbuphine consumption, and Numeric Rating Scale (NRS) pain scores in the PACU, 1, 2, 6, 12, and at 24 h postoperatively (Table 3).

**Table 3:** Postoperative outcomes

	Control group (n = 40)	ESPB group (n = 41)	P value
NRS score			
At PACU	4.5(3–5)	1(1–2)	< 0.001*
At 1 h	5(4–6)	2(2–3.5)	< 0.001*
At 2 h	5(4–6)	3(2–5)	< 0.001*
At 6 h	4(4–5)	4(3–5)	0.189
At 12 h	5(4–5)	5(3–5)	0.370
At 24 h	3(3–4)	3(2–3.5)	0.041*
Incidence of PODD, n	29/40 (73%)	4/41 (10%)	< 0.001*
Nalbuphine at 24 h	15(10–20)	10(5–10)	< 0.001*
SpO2 at 2 h	93(91–96)	98(97–99)	< 0.001*
RR (breath/minute) at 2 h	25(22–28)	16(15–18)	< 0.001*
ROX at 2 h	18(16–21)	29(27–31)	< 0.001*

^*^Denotes statistical significance

NRS: numeric rating scale, PACU: post-anesthesia care unit, PODD: postoperative diaphragmatic dysfunction, ROX: oxygenation index, RR: respiratory rate, SpO2: peripheral oxygen saturation

Multivariate logistic regression analysis revealed that ESPB group assignment was independently associated with a significantly lower risk of PODD (OR = 0.04, 95% CI: 0.01–0.16, p < 0.001). Higher insufflation pressure was also significantly associated with increased PODD risk (OR = 1.76, 95% CI: 1.02–3.04, p = 0.042). Diabetes mellitus showed a trend toward increased risk (OR = 4.80, 95% CI: 0.90–26.60, p = 0.071), though this did not reach statistical significance. Age and duration of capnoperitoneum were not significantly associated with PODD (Table 4).

**Table 4:** Multivariate regression analysis for risk factors associated with postoperative diaphragmatic dysfunction

	Odd ratio (95% CI)	P value
ESPB group vs control group	0.04(0.01–0.16)	< 0.001*
Age (years)	1.03(0.96–1.10)	0.442
DM	4.8(0.9–26.6)	0.071
Duration of capnoperitoneum (minutes)	1.00(0.94–1.07)	0.968
Insufflation pressure (cmH2O)	1.76(1.02–3.04)	0.042*

^*^Denotes statistical significance

DM: diabetes mellitus

## Discussion

This randomized controlled trial investigated the effect of bilateral ESPB on diaphragmatic function and pulmonary performance following sleeve gastrectomy in individuals with class III obesity. The findings demonstrate that the ESPB significantly reduced the incidence of PODD, preserved pulmonary function, and improved pain control compared to standard anesthesia without regional block.

The incidence of PODD was markedly lower in the ESPB group (10%) compared to the control group (73%), indicating a clinically meaningful protective effect of the block on diaphragmatic mechanics. This aligns with previous studies suggesting that interfascial plane blocks, such as ESPB, may preserve phrenic nerve activity and mitigate anesthesia-related respiratory impairment [3]. The cutoff value of < 10 mm for mean diaphragmatic excursion has been previously validated in studies assessing extubation outcomes in critically ill patients. In the context of bariatric surgery, this threshold is clinically relevant as reduced diaphragmatic mobility may contribute to postoperative hypoventilation, atelectasis, and impaired pulmonary recovery, particularly in morbidly obese individuals who are already at increased risk of respiratory complications.

Furthermore, participants in the ESPB group showed better postoperative pulmonary function, as evidenced by higher FEV1, FVC, and PEFR values. These findings are particularly relevant given the increased respiratory risk associated with class III obesity and abdominal surgery, where diminished lung volumes and altered ventilatory mechanics predispose individuals to atelectasis, hypoxemia, and delayed recovery [8]. While our study focused on the immediate postoperative period, preserved diaphragmatic function may have longer-term clinical implications. Diaphragmatic dysfunction is associated with increased risk of atelectasis, impaired gas exchange, and postoperative pneumonia, particularly in patients with class III obesity undergoing upper abdominal surgery. The improved diaphragmatic excursion and ROX index observed in the ESPB group may contribute to enhanced pulmonary mechanics and reduced respiratory complications beyond the first 24 h. Future studies with extended follow-up are warranted to evaluate the sustained impact of ESPB on pulmonary outcomes, hospital length of stay, and recovery quality.

A strong positive correlation observed between diaphragmatic excursion at 2 h and the ROX index suggests that early preservation of diaphragm function has a meaningful impact on overall respiratory efficiency. This relationship may serve as a useful indicator of postoperative respiratory status and potentially guide early interventions for respiratory support. To our knowledge, this is the first study to evaluate the ROX index in the context of bariatric surgery. The observed correlation between ROX index and diaphragmatic excursion suggests physiologic relevance and potential clinical utility. Further research is warranted to validate its prognostic value and explore its role in guiding postoperative respiratory management in obese surgical cohorts. Laparoscopic surgery results in postoperative diaphragmatic dysfunction via several pathways [2]. Moreover, class III obesity is associated with increased postoperative respiratory complications [9]. Consequently, laparoscopic sleeve gastrectomy for individuals living with class III obesity is associated with increased postoperative diaphragmatic dysfunction, with pain being the key component for such effect. Therefore, effective nerve blocks can mitigate such effects. In line with our results, recent research suggests ESPB preserves pulmonary functions in individuals undergoing laparoscopic cholecystectomy; however, they did not evaluate diaphragmatic activity [3].

Pain control was also superior in the ESPB group, with significantly lower NRS scores at PACU, 1, 2, and 24 h with reduced nalbuphine requirements within the first 24 h postoperatively. This opioid-sparing effect is desirable in individuals with obesity, in whom opioid-related respiratory depression and postoperative nausea/vomiting may be particularly detrimental [10].

Our multivariate analysis identified ESPB as a protective factor against postoperative diaphragmatic dysfunction, even after adjusting for key clinical variables. The strong association (OR = 0.04, p < 0.001) suggests a potential role for ESPB in preserving diaphragmatic function postoperatively. Elevated insufflation pressure was independently associated with increased PODD risk, underscoring the importance of intraoperative ventilatory and pressure management. Although diabetes mellitus did not reach statistical significance, the observed trend (OR = 4.80, p = 0.071) warrants further investigation, given its known impact on respiratory mechanics and wound healing. These findings support the integration of ESPB into perioperative strategies aimed at reducing respiratory complications in bariatric surgery.

Strengths of this study include its randomized design, standardized anesthetic technique, and comprehensive assessment of both functional and subjective outcomes.

## Limitations

We acknowledge some limitations to our study. First, the study was conducted at a single center with a relatively small sample size, which may limit the generalizability of the findings to broader populations living with obesity or in different surgical contexts. Second, A single operator conducted diaphragmatic examinations. In general, DE showed good interobserver reliability; however, future studies are warranted to evaluate interobserver agreement in such a cohort. Third, the evaluation was restricted to the early postoperative period (up to 24 h), and long-term outcomes, including sustained respiratory function and postoperative complications, were not assessed. Finally, sensory dermatomal assessment was not evaluated to reduce the risk of observer bias. This approach, however, may have resulted in undetected block failures. Nevertheless, all participants received effective analgesia through an appropriately implemented multimodal analgesia regimen.

## Conclusion

Bilateral ESPB in patients with class III obesity undergoing sleeve gastrectomy was associated with a significant reduction in postoperative diaphragmatic dysfunction, improved pulmonary function, and decreased nalbuphine consumption. Multivariate analysis identified ESPB as an independent protective factor, underscoring its potential role in optimizing respiratory recovery. These findings support the incorporation of ESPB into multimodal perioperative strategies for improving outcomes in high-risk obese populations.

## Data Availability

The datasets generated and/ or analyzed during the current study are available from the corresponding author on reasonable request.

## References

[1] Mostafa SF, Abdelghany MS, Abu Elyazed MM. Ultrasound-guided erector spinae plane block in patients undergoing laparoscopic bariatric surgery: a prospective randomized controlled trial. Pain Pract. 2021;21:445–53. 10.1111/papr.12975. 33295128 10.1111/papr.12975

[2] Hu J, Guo R, Li H, Wen H, Wang Y. Perioperative diaphragm dysfunction. J Clin Med. 2024. 10.3390/jcm13020519. 38256653 10.3390/jcm13020519 PMC10816119

[3] Yildiz M, Kozanhan B, Iyisoy MS, Canıtez A, Aksoy N, Eryigit A. The effect of erector spinae plane block on postoperative analgesia and respiratory function in patients undergoing laparoscopic cholecystectomy: a double-blind randomized controlled trial. J Clin Anesth. 2021. 10.1016/j.jclinane.2021.110403. 34325186 10.1016/j.jclinane.2021.110403

[4] Daes J, Morrell DJ, Hanssen A, Caballero M, Luque E, Pantoja R, et al. Paragastric autonomic neural blockade to prevent early visceral pain and associated symptoms after laparoscopic sleeve gastrectomy: a randomized clinical trial. Obes Surg. 2022;32:3551–60. 10.1007/s11695-022-06257-9. 36050617 10.1007/s11695-022-06257-9 PMC9613572

[5] Helmy MA, Mostafa L, El-zayyat NS, Ali MA, Sabry R. Impaired diaphragmatic excursion following magnesium sulfate administration in patients with preeclampsia with severe features: a prospective observational study. Int J Obstet Anesth. 2025. 10.1016/j.ijoa.2025.104347. 40101564 10.1016/j.ijoa.2025.104347

[6] Helmy MA, Magdy Milad L, Osman SH, Ali MA, Hasanin A. Diaphragmatic excursion: a possible key player for predicting successful weaning in patients with severe COVID-19. Anaesth Crit Care Pain Med. 2021. 10.1016/j.accpm.2021.100875. 33940248 10.1016/j.accpm.2021.100875 PMC8086373

[7] Adolf Helmy M, Magdy Milad L, Hasanin A, Mostafa M. The novel use of diaphragmatic excursion on hospital admission to predict the need for ventilatory support in patients with coronavirus disease 2019. Anaesth Crit Care Pain Med. 2021. 10.1016/j.accpm.2021.100976. 34748940 10.1016/j.accpm.2021.100976 PMC8570438

[8] Abdelaal GA, Eldahdouh SS, Abdelsamie M, Labeeb A. Effect of preoperative physical and respiratory therapy on postoperative pulmonary functions and complications after laparoscopic upper abdominal surgery in obese patients. Egypt J Chest Dis Tuberc. 2017;66:735–8. 10.1016/j.ejcdt.2017.10.012.

[9] Shiramoto K, Wakamatsu H, Kametani Y, Matsumoto S, Ota K, Morioka T, et al. Effect of high body mass index on postoperative pulmonary complications: a retrospective study. Ain-Shams J Anesthesiol. 2023. 10.1186/s42077-023-00312-y.

[10] Ingrande J, Lemmens HJ. Dose adjustment of anaesthetics in the morbidly obese. Br J Anaesth. 2010;105(Suppl):1. 10.1093/bja/aeq312. 21148651 10.1093/bja/aeq312

